# Supplementary material for: The early human interferon gamma response to Toxoplasma gondii is driven by Vγ9Vδ2 T-cell sensing of host phosphoantigens and subsequent NK-cell activation
Source: bioRxiv. 2025 Jun 17:2025.06.12.659293. Preprint. [Version 1] doi: 10.1101/2025.06.12.659293 (PMC12262692; doi:10.1101/2025.06.12.659293)
Supplement: Supplement 1 [file NIHPP2025.06.12.659293v1-supplement-1.pdf]

bioRxiv preprint doi: https://doi.org/10.1101/2025.06.12.659293; this version posted June 17, 2025. The copyright holder for this preprint (which was not certified by peer review) is the author/funder, who has granted bioRxiv a license to display the preprint in perpetuity. It is made available under aCC-BY 4.0 International license.

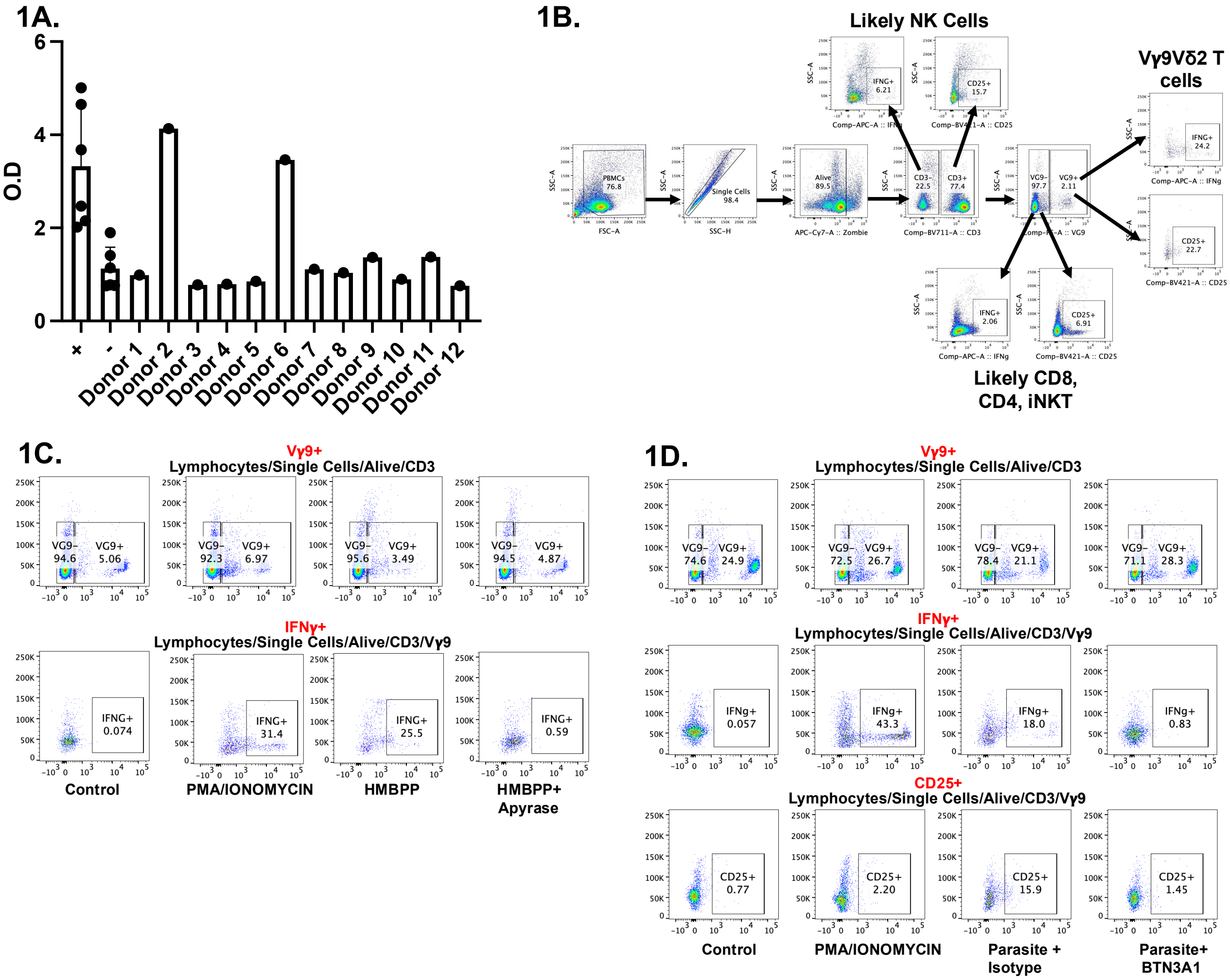

Fig S1. Serological screening of blood donors and flow-cytometric gating strategy for PBMCs. (A) Donor sera were tested for *Toxoplasma*-specific antibodies by ELISA using sonicated *Toxoplasma* lysate as the test antigen and lysates of *Sarcocystis neurona* and *Neospora hughesi* as negative controls. The optical density (OD) obtained with *Toxoplasma* lysate was divided by the mean OD of the two control lysates to generate a reactive ratio for each donor. Donors 2 and 6 were not used as they were considered *Toxoplasma* positive (**B)** Flow-cytometry gating strategy used for identifying cell populations. PBMC events were sequentially gated in FlowJo as follows: 1. Lymphocytes (SSC-A vs. FSC-A), 2. Single cells (SSC-A vs. SSC-H), 3. Viable cells (SSC-A vs. APC-Cy7::Zombie NIR), 4. CD3^{+-} (SSC-A vs. BV711::CD3), 5. Vγ9^{+-} (SSC-A vs. PE::Vγ9), 6. IFNγ^{+} (SSC-A vs. APC::IFNγ) or CD25^{+} (SSC-A vs. BV421::CD25). (**C-D**) Dot plots show the percentage of Vγ9^{+} cells within the live CD3^{+} gate and the percentage of IFNγ^{+} cells within the live CD3^{+}/Vγ9^{+} gate under the following conditions: unstimulated, (**C**) HMBPP (312 nM), HMBPP + apyrase (200 IU/ml), and PMA/ionomycin (1x). (**D)** Infection for 24 h with RHΔ*ompdc*Δ*up* parasites (MOI 0.6) with or without BTN3A1 blocking antibody or IgG1 isotype control antibody (0.1 µg/ml).

bioRxiv preprint doi: https://doi.org/10.1101/2025.06.12.659293; this version posted June 17, 2025. The copyright holder for this preprint (which was not certified by peer review) is the author/funder, who has granted bioRxiv a license to display the preprint in perpetuity. It is made available under aCC-BY 4.0 International license.

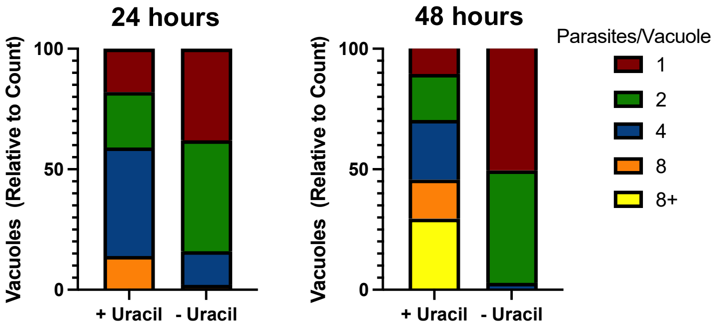

Fig S2. RHΔ*ompdc*Δ*up* parasites have impaired growth without uracil.
Parasite replication was assessed by counting parasites per vacuole in RHΔ*ompdc*Δ*up*-infected HFF monolayers cultured with or without 250 µM uracil. Parasite counts were performed at 24 hours (left) or 48 hours (right) post-infection. Samples were fixed with 3% paraformaldehyde and stained with anti-SAG2A (parasite surface marker) and DAPI. Parasites per vacuole were quantified by fluorescence microscopy.

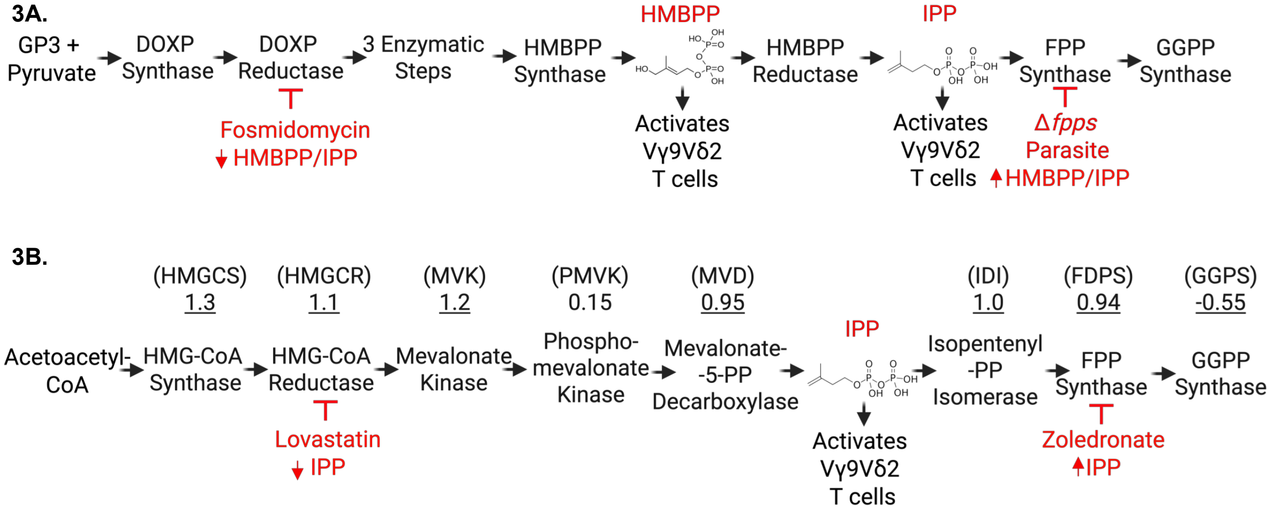

Fig S3. Comparative overview of the apicoplast 1-deoxy-D-xylulose-5-phosphate (DOXP) pathway and the host cytosolic mevalonate (MEV) pathway.
A) The non-mevalonate DOXP pathway operates in apicoplast-containing protozoa such as *Toxoplasma* and *Plasmodium spp.*, most bacteria, and in chloroplasts. Sequential reactions convert 1-deoxy-D-xylulose-5-phosphate to the phosphoantigens hydroxymethyl-butenyl pyrophosphate (HMBPP) and isopentenyl pyrophosphate (IPP). Fosmidomycin blocks DOXP reductoisomerase, halting production of both intermediates. Deletion of farnesyl-pyrophosphate synthase (Δ*fpps*) truncates the pathway downstream of IPP/HMBPP, leading to their intracellular accumulation. Created in BioRender. Rodriguez, F. (2025) https://BioRender.com/yba9idv
B) Eukaryotes synthesize IPP from acetyl-CoA via the mevalonate route. The rate-limiting step, catalysed by HMG-CoA reductase (HMGCR), is inhibited by lovastatin, preventing IPP formation.

bioRxiv preprint doi: https://doi.org/10.1101/2025.06.12.659293; this version posted June 17, 2025. The copyright holder for this preprint (which was not certified by peer review) is the author/funder, who has granted bioRxiv a license to display the preprint in perpetuity. It is made available under aCC-BY 4.0 International license.

Farnesyl-pyrophosphate synthase (FPPS) condenses IPP with dimethylallyl-PP; zoledronate inhibits FPPS, causing upstream IPP build-up. Gene IDs above each enzyme are based on RNA-seq data from the GEO dataset GSE119835 (Table S1). Numbers indicate the log2Fold change expression and underlined values denote statistical significance (adjusted p < 0.05, Benjamini-Hochberg FDR) (Table S1). Figure S3 was created in BioRender. Rodriguez, F. (2025) https://BioRender.com/yba9idv

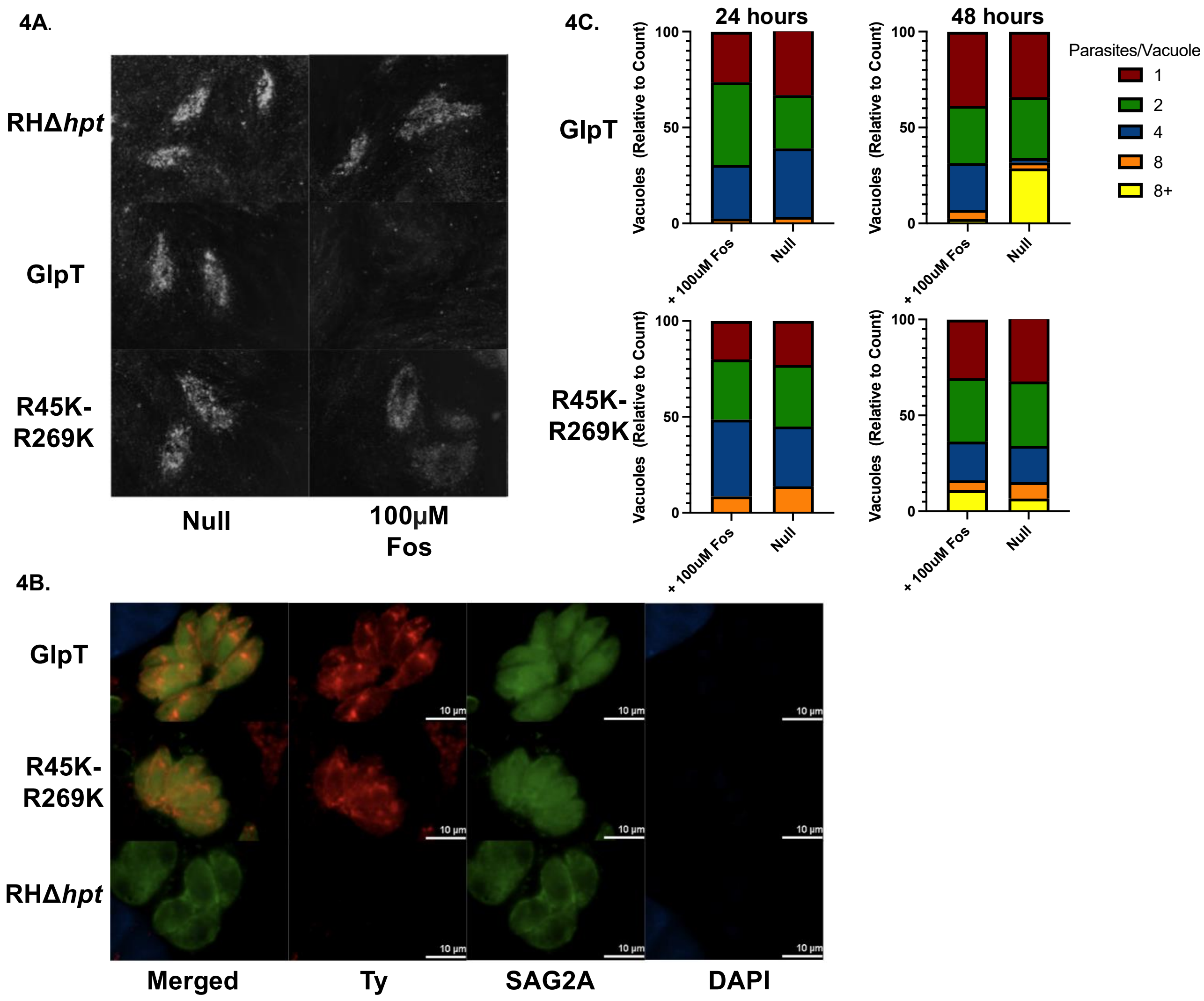

Fig S4. Fosmidomycin sensitivity in *Toxoplasma* tachyzoites requires expression of the GlpT transporter.
(**A**) Plaque assays were performed using 24-well plates with HFFs pre-incubated for an hour with or without 100 μM fosmidomycin. Cultures were then infected with 300 tachyzoites of either the parental RHΔ*hpt* strain, the GlpT transgenic line (Ty-tagged GlpT, functional glycerol-3-phosphate transporter), or the point-mutated GlpT control (R45K-R269K). Plaques were inspected after 5 days. (**B**) Immunofluorescence assays confirmed expression and localization of GlpT in the GlpT and R45K-R269K strains. Staining was performed using mouse anti-Ty, rabbit anti-SAG2A (parasite surface marker), and DAPI. Images were acquired at 100x magnification. (**C**) Parasite replication was assessed by counting parasites per vacuole at 24 and 48 hours post-infection. HFF

bioRxiv preprint doi: https://doi.org/10.1101/2025.06.12.659293; this version posted June 17, 2025. The copyright holder for this preprint (which was not certified by peer review) is the author/funder, who has granted bioRxiv a license to display the preprint in perpetuity. It is made available under aCC-BY 4.0 International license.

monolayers were pretreated with or without 100 µM fosmidomycin 1 hour prior to infection with GlpT or R45K-R269K parasites.

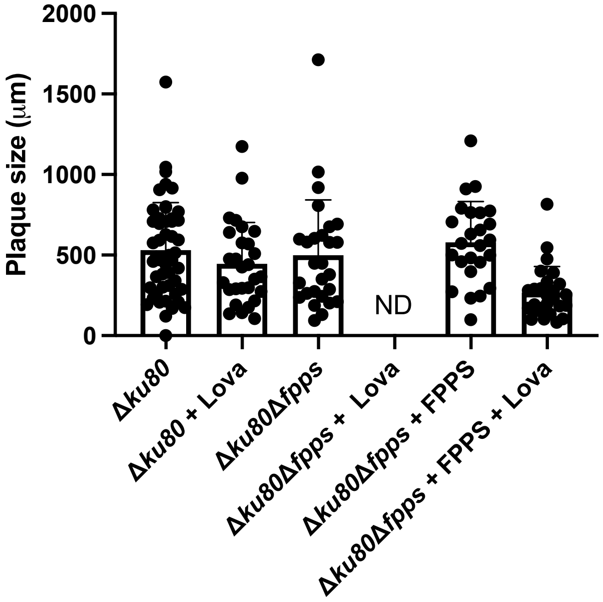

Fig S5. Lovastatin sensitivity confirms loss of FPPS activity in FPPS knockout parasites strains.
Plaque assay was performed using 24-well plates with confluent HFF monolayers pretreated for 1 hour with or without 13 µM lovastatin. Cultures were then infected with 300 tachyzoites of RHΔ*ku80* (WT), RHΔ*ku80*Δfpps (FPPS knockout), or RHΔ*ku80*Δ*fpps* + FPPS (complemented). Cultures were then analyzed with confocal microscopy to measure plaque size. FPPS knockout parasites formed plaques in drug-free medium but failed to do so in lovastatin-treated wells. In contrast, WT and complemented parasites formed normal plaques under both conditions, confirming that sensitivity to lovastatin results from loss of endogenous FPPS. Plaques were not detected (ND) for the Δ*fpps* strain in the presence of lovastatin.

Gene Symbol	Gene Name	log2FoldChange	Adjusted p-value	Pathway/Function
SQLE	squalene epoxidase	1.6	4.31E-05	Cholesterol biosynthesis
HMGCS1	3-hydroxy-3-methylglutaryl-CoA synthase 1	1.3	1.7e-05	Upstream mevalonate synthesis
MVK	mevalonate kinase	1.2	2.0e-3	Mevalonate phosphorylation
HMGCR	3-hydroxy-3-methylglutaryl-CoA reductase	1.1	3.4e-3	Cholesterol biosynthesis
FDPS	farnesyl diphosphate	0.94	1.5e-04	Isoprenoid synthesis

bioRxiv preprint doi: https://doi.org/10.1101/2025.06.12.659293; this version posted June 17, 2025. The copyright holder for this preprint (which was not certified by peer review) is the author/funder, who has granted bioRxiv a license to display the preprint in perpetuity. It is made available under aCC-BY 4.0 International license.

	synthase			
IDI1	isopentenyl-diphosphate delta isomerase 1	1.0	1.6e-2	IPP/DMAPP isomerization
MVD	mevalonate diphosphate decarboxylase	0.95	1.7e-3	Isoprenoid synthesis
PMVK	phosphomevalonate kinase	0.15	6.9e-01	Mevalonate phosphorylation
PPP2CA	protein phosphatase 2 catalytic subunit alpha	0.48	1.9e-1	PP2A catalytic subunit
ABCA1	ATP binding cassette subfamily A member 1	0.075	9.2e-1	Cholesterol efflux
IFNG	interferon gamma	7.5	2.1e-26	Cytokine
TNF	tumor necrosis factor	1.7	5.1e-3	Pro-inflammatory cytokine
IL12A	interleukin 12A	0.92	7.0e-2	Cytokine (IL-12 subunit)
IL1B	interleukin 1 beta	3.8	2.1e-17	Pro-inflammatory cytokine
BTN3A1	butyrophilin subfamily 3 member A1	-0.79	1.3e-1	Phosphoantigen recognition (Vγ9Vδ2 T cell)
BTN2A1	butyrophilin subfamily 2 member A1	-0.42	3.0e-01	BTN2 family
IL18	interleukin 18	-0.62	4.8e-01	Inflammasome-related cytokine
GGPS1	geranylgeranyl diphosphate synthase 1	-0.55	1.5E-02	Isoprenoid synthesis

bioRxiv preprint doi: https://doi.org/10.1101/2025.06.12.659293; this version posted June 17, 2025. The copyright holder for this preprint (which was not certified by peer review) is the author/funder, who has granted bioRxiv a license to display the preprint in perpetuity. It is made available under aCC-BY 4.0 International license.

Table S1. Differential expression of host genes involved in the mevalonate pathway, phosphoantigen recognition, and lipid transport in PBMCs infected with *Toxoplasma gondii*.
RNA-seq data from the GEO dataset GSE119835 were analyzed using GEO2R to compare gene expression between RH strain-infected and media-treated human PBMCs. In short, five PBMCs samples were split into three conditions: untreated, infected with RH88 (MOI 3), or infected with PRU (MOI 3). After 12 hours, RNA was extracted and sequenced (40). Differential expression was assessed by comparing RH-infected to untreated samples. Genes were selected based on known roles in the mevalonate/isoprenoid biosynthesis pathway, host phosphoantigen recognition, or lipid transport. Log2 fold change (log2FC) values represent expression differences (positive = upregulated; negative = downregulated). Adjusted p-values (Benjamini-Hochberg FDR) reflect the statistical significance of differentially expressed genes.

Primer Name	Sequence (5' to 3')	Gene/Plasmid	Use	Size	Citation
GPO-3 FW	GGGAGCAAACAGG ATTAGATACCCT	Mycoplasma 16S rRNA	Forward primer to amplify Mycoplasma contamination in cell culture via PCR	280 bp long will also see non-specific band at 100 bp	Kuppeveld et al. 1994 Applied and Environ. Microbio. 60: 149-152 (49)
MGSO RV	TGCACCATCTGTCA CTCTGTTAACCTC	Mycoplasma 16s rRNA	Reverse primer to amplify Mycoplasma contamination in cell culture via PCR	280 bp long will also see non-specific band at 100 bp	Kuppeveld et al. 1994 Applied and Environ. Microbio. 60: 149-152 (49)
GRA6 FW	ATTTGTGTTTCCGA GCAGGT	GRA6	for genotyping at GRA6 locus types I, II, or III, PCR then cut with MseI		Made in Saeij Lab
GRA6 RV	TCGCCGAAGAGTT GACATAG	GRA6	for genotyping at GRA6 locus types I, II, or III, PCR then cut with MseI		Made in Saeij Lab
GlpT FW1	ATGTTGAGTATTTTT AAACCAGCG	Glycerol 3-transporter	Glycerol 3-transporter confirmation in GlpT and R45K-R269K/ Sequencing	Used for sequencing/ 1360 bp long when using GlpT FW1 and GlpT RV1	Made in Saeij Lab
GlpT RV1	TTAGCCTCCGTTGC GTTCTT	Glycerol 3-transporter	Glycerol 3-transporter confirmation in GlpT and R45K-R269K	1360 bp long when using GlpT FW1 and GlpT RV1	Made in Saeij Lab
GlpT FW2	CGCTCTATATGCCT GCTTTCT	Glycerol 3-transporter	Glycerol 3-transporter confirmation in GlpT and R45K-R269K/ Sequencing	Used for sequencing/ 794 bp when using GlpT FW2 and GlpT RV1	Made in Saeij Lab

Table S2 Primers used to test for mycoplasma, glycerol 3-transporter, and strain type.
